# ELOVL6 in the C16/C18 Ratio Control: An Emerging Regulator of Lipid and Glucose Metabolism

**DOI:** 10.3390/biology15211998

**Published:** 2026-11-04

**Authors:** Yiyun Deng, Xinyu Su, Shiqi Wei, Shuyue Yang, Zuowei Pei

**Affiliations:** 1 Department of Geriatrics, The Fifth People’s Hospital Affiliated to Chengdu University of Traditional Chinese Medicine, Chengdu 611130, China; yun1230301@163.com; 2 Medical and Life Sciences College, Chengdu University of Traditional Chinese Medicine, Chengdu 611137, China; suxinyu_yy@163.com (X.S.); wsq_dl@163.com (S.W.); 13730702428@163.com (S.Y.)

**Keywords:** ELOVL6, fatty-acid elongation, insulin resistance, lipotoxicity

## Abstract

**Background/Objectives**: Elongation of very long chain fatty acids protein 6 (ELOVL6) is a key enzyme that catalyzes the conversion of palmitoyl-CoA (C16:0-CoA) to stearoyl-CoA (C18:0-CoA). Its product C18:0-CoA is further desaturated by stearoyl-CoA desaturase 1 (SCD1) to generate C18:1n-9-CoA. In this review, C18:0/C16:0 refers to the molar ratio of C18:0 to C16:0 within a specified lipid pool, such as acyl coenzyme A (acyl-CoA), total fatty acids, or phospholipids. It serves as a surrogate marker for ELOVL6 elongation flux under specific conditions. C18:1n-9/C18:0 refers to the oleic acid to stearic acid molar ratio. It serves as a surrogate marker for SCD1-related flux. C16:0/C18:1n-9 refers to the palmitic acid to oleic acid molar ratio. It is a composite readout influenced by both ELOVL6 and SCD1-related reactions. The “C16/C18 ratio” is used only as an umbrella term for C16/C18-related ratios. This review aims to explore how ELOVL6 modulates the balance of the C16/C18 ratio through molecular mechanisms. It also examines how this regulation contributes to glucose and lipid metabolism and to tissue-specific functions, and evaluates the potential of ELOVL6 as a therapeutic target for metabolic diseases. **Methods**: A structured literature search was conducted on recent advances regarding the enzymatic properties, transcriptional regulation, and animal model phenotypes of ELOVL6. A comprehensive synthesis was performed from molecular mechanisms to clinical translation. **Results**: In the liver, ELOVL6 mainly influences the direction of fatty acid storage and ceramide signaling. In adipose tissue, it influences insulin signal transduction by remodeling membrane phospholipid composition. In pancreatic β-cells, it affects insulin secretory function by contributing to the C16/C18 ratio. When the C16/C18 ratio becomes imbalanced, cells progress toward insulin resistance (IR), inflammation, and hepatic steatosis. **Conclusions**: Restoring the balance of the C16/C18 ratio is a potential therapeutic strategy for metabolic diseases. However, targeting ELOVL6 faces challenges such as structural biology bottlenecks and tissue-specific side effects. Future research should focus on the fine regulation of ELOVL6, combined with dietary interventions, to advance the translation of ELOVL6 from basic research to clinical intervention for metabolic diseases.

## Simple Summary

Modern diets often lead to metabolic disorders such as fatty liver disease and diabetes. These conditions are closely linked to the balance of different fat molecules in the body. This review explores the role of a specific protein called ELOVL6. It is a key factor that regulates the length of these fat molecules. We summarize recent studies to explain how this protein acts differently in various organs. These include the liver, adipose tissue, and pancreas. We found that when the balance of these fat molecules is disrupted, it triggers harmful inflammation. It also reduces the body’s response to insulin. Therefore, restoring this balance is a promising strategy to treat metabolic diseases. However, developing related drugs remains challenging. This is due to potential side effects in different organs. Future treatments may need to combine precise targeted drugs with dietary interventions to improve human health.

## 1. Introduction

### 1.1. Research Background

Lipid metabolism is the process by which the body digests, absorbs, synthesizes, degrades, and transports lipid molecules. Its primary role is to efficiently store excess chemical energy in adipose tissue, serving as an energy reserve to cope with nutritional fluctuations [1]. However, recent studies have revealed that lipid metabolism is not limited to energy storage. The carbon chain length and degree of saturation of fatty acids directly determine their physicochemical properties. This in turn influences many biological functions, including the physicochemical properties of cell membranes [2,3], signal transduction efficiency [4,5], and inflammatory responses [6,7].

During the regulation of lipid metabolism, the elongation of very long chain fatty acids (ELOVL) family has drawn considerable attention for its key role in determining the carbon chain length of fatty acids. The mammalian ELOVL family consists of seven members, each with distinct substrate specificities and tissue distributions. Their functions are closely associated with the onset and progression of various metabolic diseases [1,8] (Table 1). As an important member of the ELOVL family, ELOVL6 is the key enzyme responsible for elongating palmitoyl-CoA (C16:0-CoA) to stearoyl-CoA (C18:0-CoA) and palmitoleoyl-CoA (C16:1n-7-CoA) to vaccenic-CoA (C18:1n-7-CoA) [8,9,10]. In addition, C18:0-CoA can serve as a substrate for SCD1 to be desaturated into oleoyl-CoA (C18:1n-9-CoA) [8]. Therefore, ELOVL6 and functionally coupled enzymes such as SCD1 are important targets for the regulation of lipid metabolism and disease intervention [11,12]. In this review, we aim to elucidate the central role of ELOVL6 in the elongation of saturated fatty acids (SFAs) and monounsaturated fatty acids (MUFAs). We also address its pathophysiological significance in insulin resistance (IR), obesity, and metabolic dysfunction-associated steatotic liver disease (MASLD).

### 1.2. Literature Search Strategy

For this narrative review, we searched databases including PubMed/MEDLINE, Scopus and Web of Science. The search covered the period from September 2001 to May 2026. The main search terms included: ELOVL6, fatty acid elongase 6, lipid metabolism, insulin resistance, fatty liver, non-alcoholic fatty liver disease, obesity, lipotoxicity, and palmitic acid. The included literature types comprised original experimental studies, clinical trials, and authoritative reviews. Articles lacking sufficient mechanistic evidence and case reports were excluded. Literature screening was conducted in two stages. We first performed an initial screening of titles and abstracts. This was followed by a full-text evaluation based on predefined criteria. As a narrative review, the synthesis in this article reflects the authors’ selection and interpretation of existing evidence. It is not a systematic search with quantitative synthesis. Therefore, the possibility of selection bias cannot be completely ruled out [13].

**Table 1:** Substrate preferences, physiological functions, and biological significance of the mammalian ELOVL family.

Isoform	Preferred Substrate(s)	Tissue Distribution (Major)	Key Physiological/Biological Function	Reference
ELOVL1	Preferentially elongates C18-C26 saturated/monounsaturated acyl-CoAs, with highest activity for C20–C22	Skin (epidermis)	Maintains skin barrier function and sphingolipid levels; involved in VLCFA incorporation into membrane lipids, affecting membrane stability/fluidity	[14,15]
ELOVL2	Mainly elongates C20–C22 PUFAs	Testis (highest); liver; brain, adipose tissue	Regulator of PUFA synthesis; loss of function causes ER stress, mitochondrial dysfunction, and accelerates aging (hair loss, decreased bone density, cognitive decline)	[16,17]
ELOVL3	Preferentially elongates C18 acyl-CoA (especially C18:0); also acts on C16–C22 saturated/monounsaturated acyl-CoAs	BAT; liver; skin (sebaceous glands)	Involved in BAT recruitment; long-term cold exposure maintains its high expression	[18]
ELOVL4	Synthesizes ≥ C28 VLC-PUFAs, including n-3 VLC-PUFAs	Retina (photoreceptors); skin; brain; testis	Essential for vision and photoreceptor cell integrity; maintains skin homeostasis and barrier; mutations are associated with ichthyosis	[19]
ELOVL5	Involved in C18–C20 PUFA elongation (low activity for C22)	Various human tissues; highest in testis and epididymis, correlated with high 22:5n-6 content	Key elongase in activated T cells, responsible for elongating 18/20-carbon PUFAs to support membrane lipid remodeling; does not directly regulate T cell proliferation or activation	[20]
ELOVL6	Converts saturated and monounsaturated C16 fatty acids to C18 species (highest activity for C16:0)	Widely expressed, especially in lipid-rich tissues: BAT, WAT, liver	Involved in lipogenesis and fatty acid quality control; *Elovl6* knockout improves insulin resistance in the context of obesity; metabolic phenotype is background-dependent	[21,22]
ELOVL7	Associated with the metabolism of SVLFAs and their derivatives	Expressed in most tissues, except heart and skeletal muscle	Promotes tumor growth and survival in prostate/lung cancer; drives ferroptosis in podocytopathy (knockout alleviates kidney injury)	[23,24]

Abbreviations: Acyl-CoA, acyl coenzyme A; BAT, brown adipose tissue; ELOVL, elongation of very long chain fatty acids protein; ER, endoplasmic reticulum; PUFA, polyunsaturated fatty acid; SVLFA, saturated very long-chain fatty acid; VLC-PUFA, very long-chain polyunsaturated fatty acid; VLCFA; very long-chain fatty acid; WAT, white adipose tissue.

## 2. Molecular Characteristics and Catalytic Mechanism of ELOVL6

### 2.1. Enzymatic Function

**Figure 1:**
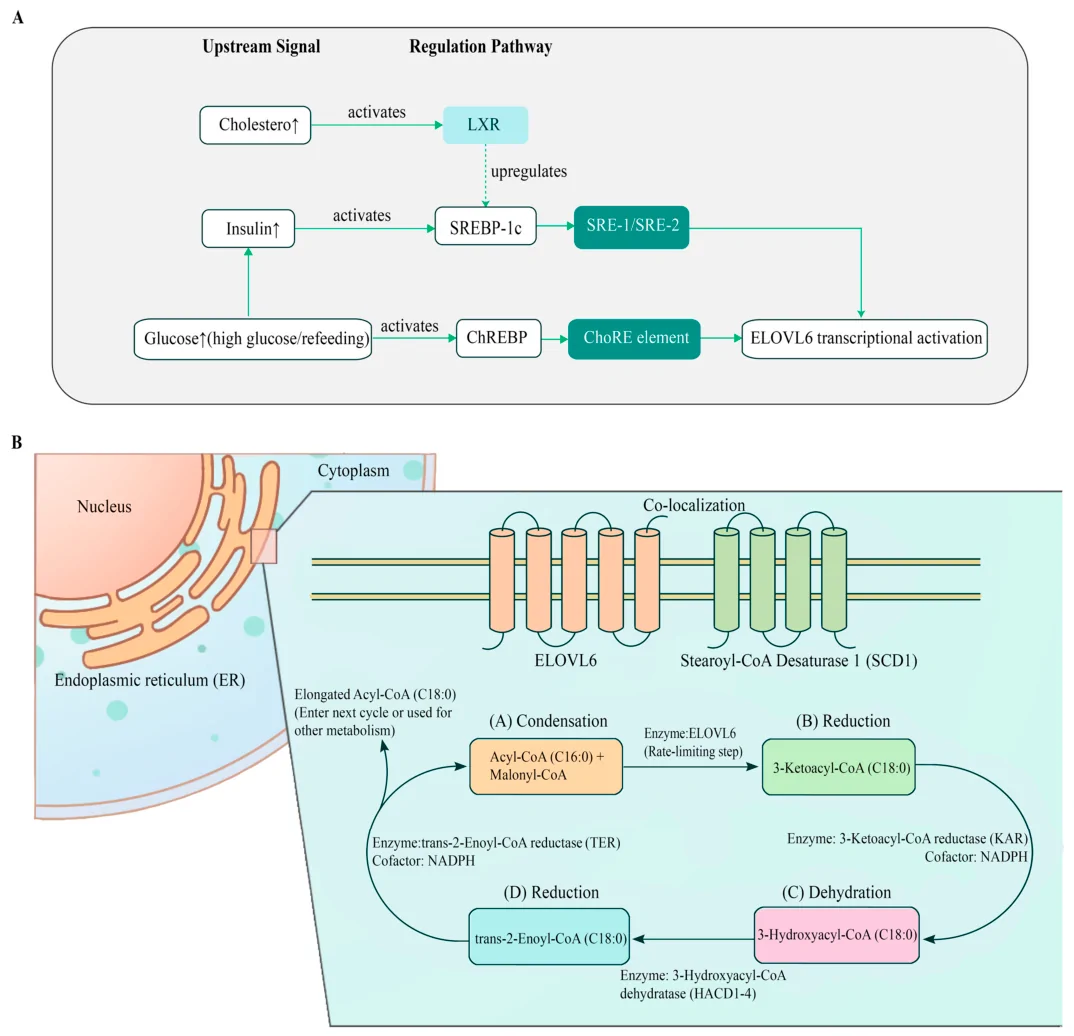
Regulatory Network and Catalytic Cycle of ELOVL6. (**A**) Elevated cholesterol activates liver X receptor (LXR). LXR indirectly promotes ELOVL6 transcription by upregulating sterol regulatory element-binding protein 1c (SREBP-1c) (dashed arrow). Elevated insulin directly activates SREBP-1c. SREBP-1c then binds to sterol regulatory elements (SRE-1/SRE-2) in the ELOVL6 promoter. Glucose (hyperglycemia/refeeding) activates carbohydrate response element-binding protein (ChREBP). ChREBP binds to the carbohydrate response element (ChoRE). SREBP-1c and ChREBP cooperatively activate ELOVL6 transcription. (**B**) ELOVL6 colocalizes with stearoyl-CoA desaturase 1 (SCD1) on the endoplasmic reticulum (ER) membrane. ELOVL6 and downstream elongases together catalyze a four-step reaction sequence. This converts C16:0-CoA to C18:0-CoA through condensation, reduction, dehydration, and a second reduction. ELOVL6 catalyzes the initial condensation step. It is the rate-limiting enzyme of this elongation cycle. This step is rate-limiting because it determines substrate specificity and is irreversible. The subsequent reduction, dehydration, and second reduction are catalyzed by different downstream enzymes, such as KAR, HACD, and TER. SCD1 desaturates C18:0-CoA to generate C18:1n-9-CoA.

ELOVL6 is a microsomal enzyme located on the endoplasmic reticulum (ER). It catalyzes fatty acid elongation through a cycle that uses malonyl-CoA as a two-carbon donor and proceeds via four sequential reactions: condensation, reduction, dehydration, and a second reduction [8] (Figure 1). ELOVL6 primarily elongates saturated and monounsaturated fatty acyl-CoAs (acyl coenzyme A) with chain lengths of 12, 14, and 16 carbons [2,11,25]. In terms of substrate preference, ELOVL6 displays the highest catalytic activity toward C16:0-CoA, converting it to C18:0-CoA. It also efficiently converts palmitoleoyl-CoA (C16:1n-7-CoA) to vaccenoyl-CoA (C18:1n-7-CoA) [11,26].

Although the enzymatic function of ELOVL6 has been characterized, understanding how it achieves such strict substrate specificity requires structural information. The crystal structure of human ELOVL7 (PDB: 6Y7F) provides a framework for understanding the molecular architecture of the ELOVL family. ELOVL7 adopts an inverted transmembrane barrel structure. It encloses a substrate-binding tunnel approximately 35 Å long, with the catalytic center deeply buried within the membrane. Mechanistically, catalysis proceeds via the formation of a covalent acyl-enzyme intermediate involving a conserved HxxHH motif. Crucially, while the catalytic core is conserved, the residues forming the acyl-chain binding pocket are highly divergent among ELOVL isoforms. This sequence variability, particularly in the TM6–TM7 loop and the N-terminal region of TM7, is thought to alter the size and shape of the tunnel. This consequently determines isoform-specific chain-length preference. Based on this structural logic, we speculate that the substrate-binding tunnel of ELOVL6 is more restricted than that of ELOVL7. This is consistent with its specific preference for C12-C16 acyl-CoAs [27].

### 2.2. Subcellular Localization

ELOVL 6 is a multi-pass transmembrane protein localized to the ER, with its active site facing the cytoplasmic side [11]. Both immunofluorescence and subcellular fractionation experiments have confirmed that ELOVL6 highly colocalizes with ER marker proteins [8,9]. ELOVL6 localizes to the ER through its own transmembrane domains and does not depend on other binding partners [9]. This topology confines the elongation reaction to the cytoplasmic side of the ER membrane, allowing newly synthesized long-chain acyl-CoAs to directly enter downstream metabolic pathways [8].

In the broader metabolic network, ELOVL6 and SCD1, both located on the ER membrane, form a close functional coupling [1,28]. The overlapping localization of the two enzymes on the ER membrane ensures efficient substrate channeling between desaturation and elongation reactions [1,29]. Functionally, SCD1 converts C16:0-CoA and C18:0-CoA to the monounsaturated fatty acyl-CoAs, C16:1n-7-CoA and C18:1n-9-CoA, respectively [26,30,31]. Together, they influence the intracellular SFA/MUFA ratio and constitute a key node linking de novo lipogenesis (DNL) to metabolic regulation [32].

### 2.3. Transcriptional Regulation

*Elovl6* expression is mainly regulated by three transcriptional pathways. The sterol regulatory element-binding protein-1c (SREBP-1c) pathway and the carbohydrate-responsive element-binding protein (ChREBP) pathway act through direct transcriptional regulation, whereas the liver X receptor (LXR) pathway primarily functions via indirect regulation. These three pathways respond to insulin, glucose, and cholesterol signals, respectively.

The SREBP-1c pathway activates *Elovl6* transcription in response to insulin signaling. The *Elovl6* promoter contains two sterol regulatory elements (SRE-1 and SRE-2) [33]. Both are essential for transcriptional activation, and the proximal SRE-1 has higher affinity for SREBPs and serves as the primary binding site [33,34,35]. Insulin enhances nuclear SREBP-1c activity, thereby upregulating *Elovl6* expression.

The ChREBP pathway primarily responds to glucose signals and adjusts metabolic responses as blood glucose levels fluctuate. Upon glucose stimulation, ChREBP translocates to the nucleus, binds the carbohydrate response element (ChoRE) in the *Elovl6* promoter, and activates transcription [4,35]. Refeeding after fasting strongly induces *Elovl6* expression, and this effect is abolished in MLX interacting protein-like (*Mlxip1*) knockout mice. This indicates that ChREBP pathway regulates *Elovl6* in response to nutritional status. Furthermore, ChREBP and SREBP-1c pathway cooperatively regulate *Elovl6* transcription, with the SREBP-1c pathway playing the dominant role [35].

The LXR pathway is an important nuclear receptor in the lipid metabolism regulatory network, but direct evidence for its regulation of *Elovl6* is relatively limited. LXR agonists only weakly induce *Elovl6* in rat primary hepatocytes, and this effect depends mainly on upregulating SREBP-1c expression rather than direct binding of LXR pathway to the *Elovl6* promoter. Therefore, LXR pathway regulates *Elovl6* primarily through the SREBP-1c pathway [36].

## 3. ELOVL6 and Lipid Metabolism: From Lipid Droplets to Lipotoxicity

### 3.1. The Central Role of the C16/C18 Ratio

ELOVL6 is a key enzyme that controls the elongation of C16-CoAs to C18-CoAs, and its activity influences the C16/C18 ratio in the ER acyl-CoA pool [1,11]. When ELOVL6 is highly expressed, C16:0-CoA and C16:1n-7-CoA are efficiently converted to C18:0-CoA and C18:1n-7-CoA, lowering the C16/C18 ratio (increasing the C18:0/C16:0 ratio). When ELOVL6 is inhibited or deficient, C16:0 and C16:1n-7 accumulate and the C16/C18 ratio rises (the C18:0/C16:0 ratio falls) [37]. Given that the C16:0/C18:1n-9 ratio incorporates SCD1 activity, it more comprehensively reflects the balance between SFAs and MUFAs in the measured lipid pool [28].

C16:0 is a lipotoxic saturated fatty acid [10,31]. It can induce ER stress, inflammatory responses, and mitochondrial dysfunction in hepatocytes and pancreatic β-cells, ultimately leading to apoptosis [7,31,38]. Compared with C16:0, free C18:0 and C18:0 stored in triglycerides (TG) are less lipotoxic. They may even exert a relatively protective effect. However, this conclusion cannot be extrapolated to C18:0-ceramide. C18:0-ceramide can affect insulin signal transduction (see Section 3.2 and Section 4.1 for details) [7,39].

When the specified C16/C18 ratio becomes imbalanced in the relevant lipid pool, cells progress toward IR, inflammation, and hepatic steatosis. In the context of MASLD, patient-derived exosomes show a significantly elevated C16:0/C18:1n-9 ratio. Loading C18:1n-9 onto MASLD patient-derived exosomes restores the C16:0/C18:1n-9 ratio, and even with high C16:0 levels still present, hepatocyte viability and proliferation are recovered [7]. This indicates that the imbalanced fatty acid ratio carried by exosomes can itself serve as a signaling molecule. It reflects the survival state of recipient cells. In a drug-induced liver injury model, repeated administration of amiodarone suppresses ELOVL6 and SCD1 expression, leading to C16:0 accumulation, reduced C18 MUFAs, an elevated C16/C18 ratio, and ultimately hepatocyte apoptosis [40]. These findings indicate that an imbalanced C16/C18 ratio is an important factor in the development and progression of metabolic diseases. Restoring the C16/C18 balance, particularly by correcting the C16:0/C18:1n-9 ratio in the relevant lipid pool, represents a potential therapeutic strategy for lipid metabolism disorders.

### 3.2. Triacylglycerol Synthesis and Storage

Triacylglycerol synthesis is accomplished primarily through the Kennedy pathway. After activation by acyl-CoA synthetase, both C18:0-CoA and C18:1n-9-CoA can serve as substrates for diacylglycerol acyltransferase (DGAT). ELOVL6 contributes to the supply of C18-CoA in the DGAT substrate pool. It thereby indirectly regulates the rate of TG synthesis and its fatty acid composition [41,42]. In this process, glycerol-3-phosphate (G3P) undergoes four sequential enzymatic reactions catalyzed by glycerol-3-phosphate acyltransferase (GPAT), 1-acylglycerol-3-phosphate acyltransferase (AGPAT), phosphatidic acid phosphatase (Lipin), and DGAT, generating lysophosphatidic acid (LPA), phosphatidic acid (PA), diacylglycerol (DAG), and finally TG.

Whether high *ELOVL6* expression plays a compensatory protective role in hepatic steatosis remains a controversial issue in the field [1]. Some studies suggest that the strong lipotoxicity of C16:0 is the primary culprit in liver injury, and therefore *ELOVL6* overexpression represents a compensatory protective response [6]. However, other studies have found that the elongation products of ELOVL6 can also cause cellular damage [5].

Under normal physiological conditions, C18:0 mainly enters the TG storage pathway [42], and *ELOVL6* expression thus plays a compensatory protective role. However, under pathological conditions, ELOVL6 overexpression expands the C18:0-CoA pool. When this pool exceeds the storage capacity of TG, C18:0-CoA is diverted into other pathogenic pathways. One of these is the inflammatory pathway. C18:0-CoA can be hydrolyzed to free C18:0. Free C18:0 activates the NLR family pyrin domain containing 3 (NLRP3) inflammasome, promotes Interleukin-1 β (IL-1β) release, and exacerbates the inflammatory response [6,43]. Studies have shown that inflammatory signals can drive the elongation of C16:0-CoA to C18:0-CoA in Kupffer cells. The generated C18:0-CoA, or the free C18:0 derived from it, further aggravates inflammation by activating the NLRP3 inflammasome. This forms a vicious cycle of the disease. Another pathway is the ceramide pathway. Excess C18:0-CoA condenses with sphingosine via ceramide synthase 4 (CerS4) to generate C18:0-ceramide. This ceramide directly inhibits hepatic insulin signaling through the protein phosphatase 2A inhibitor-2 (I2PP2A)-protein phosphatase 2A (PP2A)-protein kinase B (Akt) pathway [5].

Therefore, the role of ELOVL6 in hepatic lipid metabolism is highly context-dependent. Its function is dynamically governed by the cellular lipid environment. Specifically, the balance between TG storage capacity and the activity of pathways such as CerS4 and NLRP3 determines whether ELOVL6 acts as a protective buffer or a pathogenic driver.

### 3.3. Membrane Lipidomics

Membrane lipid composition directly influences the physical properties of the membrane and the function of membrane proteins. ELOVL6 alters the chain-length composition of fatty acids in membrane phospholipids, thereby exerting an important effect on membrane physical properties [44]. An increase in the chain length of fatty acids in membrane phospholipids makes the membrane thicker and more rigid, whereas a decrease in chain length makes the membrane thinner and more fluid [3]. Therefore, ELOVL6 can maintain normal membrane thickness and fluidity by regulating the proportion of fatty acids in membrane phospholipids [2]. However, it is worth noting that membrane thickness and fluidity are also affected by fatty acid unsaturation and lipid composition. Specifically, higher unsaturation usually increases membrane fluidity. Changes in other lipid species can further modulate these physical properties. Therefore, ELOVL6-mediated changes in carbon chain length act in concert with these broader lipidomic factors to maintain optimal membrane thickness and fluidity.

Knockdown of hormone-sensitive lipase (HSL) in human adipocytes enhances ChREBP activity, which upregulates *ELOVL6* expression and increases the C18:1n-9 content in phospholipids. This is accompanied by enhanced insulin-stimulated phosphorylation of insulin receptor substrate-1 (IRS-1) and Akt. Conversely, when *ELOVL6* is knocked down, or when phospholipid composition is restored by C18:1n-9 supplementation, the enhancement of insulin signaling is reversed. These findings demonstrate that ELOVL6 can influence insulin signaling by modulating the C16:0/C18:1n-9 ratio in membrane phospholipids [4].

## 4. Elovl6 in Glucose Homeostasis and Insulin Signaling

### 4.1. C16:0-Mediated Molecular Pathways of Insulin Signaling Inhibition

The intracellular C18:0/C16:0 acyl-CoA ratio has been used as a surrogate index of ELOVL6-mediated elongation and is associated with downstream insulin signaling [1]. Clinical studies have confirmed that lower estimated ELOVL6 activity, inferred from fatty-acid ratios, is associated with higher insulin sensitivity [5,45]. Therefore, the C18:0/C16:0 ratio is also used as an important biomarker for assessing insulin sensitivity [45].

The regulation of insulin signaling by ELOVL6 is mainly associated with the following mechanisms: the diacylglycerol/protein kinase Cε (DAG/PKCε) pathway and the ceramide/PP2A pathway [46,47].

In the DAG/PKCε pathway, C16:0 is esterified to fatty acyl-CoA and enters the glycerolipid synthesis pathway. The supply of C16:0 promotes the production of the intermediate DAG. However, DAG accumulation is tightly controlled by glycerolipid flux. Specifically, this depends on the balance between DAG synthesis and its further conversion to TG by DGAT. Furthermore, PKCε activation is not triggered by total cellular DAG. Instead, it is mediated by the accumulation of specific DAG species in specific membrane compartments. When these specific DAG species accumulate, they act as potent endogenous PKCε activators. This leads to phosphorylation at the Thr1160 site of the insulin receptor, thereby impairing downstream insulin signaling [46].

In the ceramide/PP2A pathway, C16:0-CoA can condense with sphingosine/dihydrosphingosine via ceramide synthase 6 (CerS6) to generate C16:0-ceramide [47]. C16:0-ceramide may affect insulin signaling through mechanisms such as directly binding to PP2A [5,48,49].

**Figure 2:**
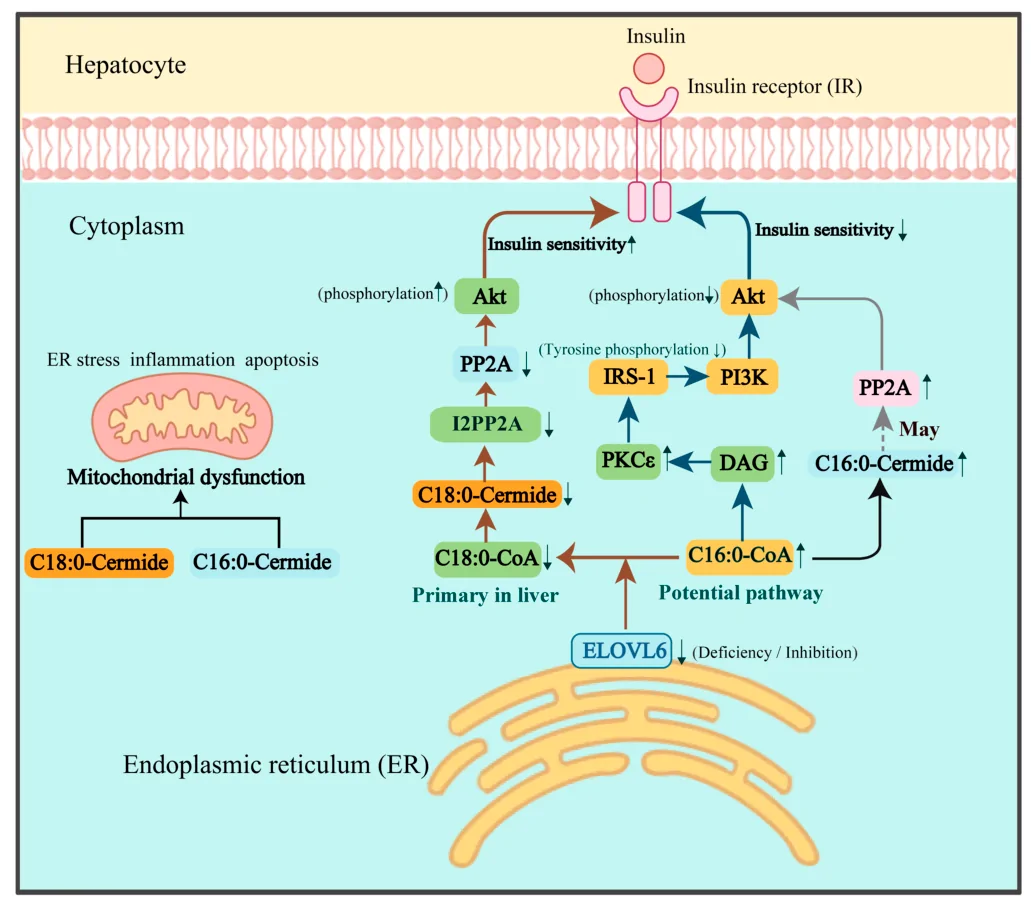
Effects of ELOVL6 deficiency or inhibition on insulin signaling and mitochondrial function in hepatocytes. ELOVL6 deficiency leads to a decrease in C18:0-CoA and its downstream product C18:0-ceramide (Primary in liver). The reduced C18:0-ceramide fails to interfere with I2PP2A, allowing I2PP2A to inhibit PP2A. This leads to decreased PP2A activity and increased Akt phosphorylation, thereby enhancing insulin sensitivity. Concurrently, accumulated C16:0-CoA acts through the DAG/PKCε pathway (in which specific DAG species in specific membrane compartments, not total DAG, activate PKCε) to impair IRS-1 tyrosine phosphorylation, and through the C16:0-ceramide pathway (Potential pathway) to activate PP2A, both contributing to reduced Akt phosphorylation and decreased insulin sensitivity. Furthermore, both C18:0-ceramides and C16:0-ceramides can induce mitochondrial dysfunction, leading to ER stress, inflammation, and apoptosis.

Notably, C16:0-CoA can be elongated by ELOVL6 to C18:0-CoA. C18:0-CoA is then converted by CerS4 in the ER to generate C18:0-ceramide. CerS4 uses C18:0-CoA, rather than free C18:0, as its acyl donor. In this process, C18:0-ceramide interferes with the binding of PP2A to I2PP2A. This relieves the inhibition of PP2A by I2PP2A and enhances PP2A activity. Activated PP2A dephosphorylates Akt and thereby blocks downstream insulin signaling. In the liver, C16:0-ceramide appears to regulate the I2PP2A-PP2A axis less strongly than C18:0-ceramide. Under high-sucrose diet (HSD) or high-fat/high-sucrose (HFHS) diet conditions, C18:0-ceramide appears to be the predominant effector of the PP2A/Akt axis in the liver. Accordingly, in these dietary models, ELOVL6 inhibition reduces C18:0-ceramide and improves insulin sensitivity [5] (Figure 2).

### 4.2. Phenotypic Analysis of Elovl6^−/−^ Mice

To date, most phenotypic analyses of *Elovl6*^−/−^ mice have shown that the metabolic effects are largely diet-dependent and tissue-specific. Unless otherwise specified, all mice discussed in this section are on a C57BL/6J background (backcrossed for at least six generations).

Under high-fat diet (HFD) conditions, whole-body *Elovl6*^−/−^ mice gained body weight and developed hepatic steatosis to a similar degree as wild-type (WT) mice, but their plasma insulin levels were significantly lower, suggesting improved insulin sensitivity [22]. However, in liver-specific knockout (LKO) mice, the degree of hepatic steatosis and IR did not differ from that of controls [5]. This indicates that under HFD, endogenous fatty acid synthesis is not the dominant pathway, and the loss of hepatic ELOVL6 can be compensated by peripheral tissues.

Under an HSD, the dietary context potently activates the ChREBP and SREBP-1c pathways, driving robust hepatic DNL. In this dietary context, liver-specific *Elovl6*^−/−^ mice showed significantly improved insulin sensitivity, along with reduced hepatic C18:0-ceramide content, decreased PP2A activity, and restored Akt phosphorylation [5].

Under a high-fat high-sucrose (HFHS) diet, whole-body knockout mice not only displayed more pronounced improvements in insulin sensitivity than under HFD alone, but also exhibited enhanced glucose-stimulated insulin secretion (GSIS) [50]. When whole-body knockouts were crossed onto an *ob/ob* (leptin-deficient) background, their hyperglycemia was also significantly alleviated even on a normal chow diet [22,39].

Taken together, the improvement in IR primarily is associated with DNL-dependent remodeling of the hepatic C18:0-CoA/C16:0-CoA ratio and downstream C18:0-ceramide, independent of changes in body weight or hepatic lipid content [5,12]. HFHS and HSDs potently activate the ChREBP and SREBP-1c pathways, driving the liver to synthesize fatty acids such as C16:0 [35,51]. When ELOVL6 is knocked out, C16:0 accumulates substantially while C18:0 becomes depleted. Under these conditions, the PP2A/Akt pathway is suppressed, and insulin sensitivity improves [5,12]. In contrast, under HFD conditions, C16:0 accumulation is limited and the effect on these axes is not apparent, which explains the lack of efficacy of LKO. Therefore, the metabolic effects of ELOVL6 inhibition depend on the dietary background and tissue specificity. Under diets that strongly drive DNL, such as HSD, both whole-body and LKO models improve insulin sensitivity by preventing ceramide accumulation. Conversely, under HFD, LKO has minimal effect.

### 4.3. ELOVL6 and Pancreatic β-Cell Function

The survival and secretory function of pancreatic β-cells depend on the balance between C16:0 and C18:1n-9 fatty acids regulated by ELOVL6 [11,50,52].

In *db/db* mice (on a C57BLKS/J background, a model of type 2 diabetes mellitus (T2DM) caused by leptin receptor deficiency), *Elovl6*^−/−^ mice showed a significant increase in β-cell mass, characterized by increased proliferation and reduced apoptosis, accompanied by adaptive increases in insulin and improved glycemic control [52,53]. Mechanistically, in wild-type *db/db* islets, C18:0-CoA is converted by SCD1 into C18:1n-9-CoA, which is markedly elevated. Excess C18:1n-9-containing lipids induce endoplasmic reticulum stress and inflammation in β-cells, leading to apoptosis and impaired insulin secretion. After *Elovl6* deletion, ELOVL6-derived C18:0-CoA and its downstream product C18:1n-9-CoA decrease, thereby alleviating the stress burden imposed on β-cells by excessive MUFA accumulation [52]. In the ZDF (Zucker Diabetic Fatty) rat model of overt diabetes, the expression of *Scd1* and *Elovl6* in islets declines significantly as the disease progresses. This reduces the conversion of C16:0-CoA to C18:0-CoA and of C18:0-CoA to C18:1n-9-CoA, leading to accumulation of lipotoxic saturated fatty acid species such as C16:0 and C16:0-derived lipids, which intensifies β-cell damage [54]. In the former study, reducing C18:1n-9 alleviated the adverse effects of excess MUFA, whereas in the latter, C16:0 accumulation accelerated SFA-induced lipotoxic injury. Together, these two studies illustrate the bidirectional effects of C16:0/C18:1n-9 ratio changes under different pathological states.

Furthermore, in an HFHS diet-induced IR model, islet hypertrophy was suppressed in *Elovl6*^−/−^ mice, and GSIS was enhanced [50]. Mechanistically, the enhanced GSIS was associated with an increased ATP/ADP ratio and suppressed activating transcription factor 3 (ATF-3) expression. ATF-3 is a stress-responsive transcription factor, and its elevated expression is closely linked to β-cell apoptosis and dysfunction. *Elovl6* deficiency reduced the stress burden of β-cells by suppressing ATF-3, thereby preserving their secretory capacity.

In summary, through its conversion of C16:0-CoA to C18:0-CoA and the subsequent SCD1-mediated generation of C18:1n-9-CoA, ELOVL6 helps maintain the balance between C16:0-containing and C18:1n-9-containing lipids in β-cells. Therefore, the effects of ELOVL6 on pancreatic β-cells are bidirectional and stage-dependent. The differential effects of *Elovl6* knockout indicate that we must accurately identify the disease stage and the specific lipid species driving lipotoxicity. This identification is a prerequisite for ELOVL6-targeting strategies in β-cells. However, the concept of stage-dependent ELOVL6 inhibition is still based largely on indirect comparisons between the *db/db* and ZDF models, and direct evidence from time-course interventions within a single model is lacking. Its clinical feasibility remains to be further validated [1,11]. A sumary of the experimental evidence discussed above is provided in Table 2.

**Table 2:** Summary of experimental evidence for ELOVL6 in tissue-specific and diet-dependent metabolic regulation.

Tissue	Model/Genotype	Intervention/Diet	Fatty-Acid Species/Ratio Measured	Metabolic Outcome	Reference
Liver	Whole-body *Elovl6*^−/−^ mice	HFD	C18:0/C16:0 ratio ↓; PKCε activity ↓	Improved insulin sensitivity, lower plasma insulin	[22]
Liver	Liver-specific *Elovl6*^−/−^ mice	HFD	C16:0 accumulation, C18:0 depletion	No significant difference in hepatic steatosis or IR	[5]
Liver	Liver-specific *Elovl6*^−/−^ mice	HSD/HFHS	C18:0-ceramide ↓, PP2A activity ↓	Significantly improved insulin sensitivity and Akt phosphorylation	[5]
Liver	Whole-body *Elovl6*^−/−^ mice	Atherogenic high-fat diet	C16:0/C18:1n-9 ratio ↓	Improved inflammation, oxidative stress, and fibrosis (MASH model)	[6]
Liver	Whole-body *Elovl6*^−/−^ mice on *Ldlr*^−/−^ background	Lithogenic diet	(Bile acid homeostasis)	Promoted cholesterol crystallization and gallstone formation (Risk)	[55]
Pancreatic β-cells	Whole-body *Elovl6*^−/−^ mice on *db/db* background	Normal chow	C18:0 and C18:1n-9 ↓	Increased β-cell mass, reduced apoptosis, improved GSIS	[52]
Pancreatic β-cells	ZDF rats	Overt diabetes progression	C16:0 accumulation ↑ (due to Scd1/Elovl6 decline)	Exacerbated lipotoxicity and β-cell damage	[54]
WAT	HSL-knockdown human adipocytes	In vitro	Phospholipid C18:1n-9 ↑	Enhanced insulin-stimulated IRS-1 and Akt phosphorylation	[4]
BAT	Whole-body *Elovl6*^−/−^ mice	Cold exposure	(Membrane fatty acid remodeling)	Impaired mitochondrial ETC components, reduced thermogenic capacity	[28]

Note: ↑ indicates an increase; ↓ indicates a decrease. Abbreviations: HFD, high-fat diet; HFHS, high-fat high-sucrose diet; HSD, high-sucrose diet; IR, insulin resistance; GSIS, glucose-stimulated insulin secretion; WAT, white adipose tissue; BAT, brown adipose tissue; MASH, metabolic dysfunction-associated steatohepatitis; ZDF, Zucker diabetic fatty; PP2A, protein phosphatase 2A; Akt, protein kinase B; Ldlr, low density lipoprotein receptor; HSL, hormone-sensitive lipase; IRS-1, insulin receptor substrate 1; ETC, electron transport chain.

## 5. Tissue-Specific Functional Differences in ELOVL6

ELOVL6 displays marked tissue specificity. This arises from its regulation by distinct transcriptional networks in different tissues. It also results from the differing responses of each tissue to ELOVL6 catalytic activity.

### 5.1. Regulation of ELOVL6 Expression in the Liver

The liver is the most extensively studied tissue for ELOVL6 function. In the liver, the ELOVL6 product C18:0-CoA is the direct precursor of C18:0-ceramide. C18:0-ceramide can negatively regulate insulin signaling through the I2PP2A-PP2A-Akt pathway [5]. This mechanism has not been shown to be equally important in other tissues. In patients with MASLD or metabolic dysfunction-associated steatohepatitis (MASH), hepatic *ELOVL6* expression correlates positively with the degree of steatosis and serum alanine aminotransferase (ALT) levels [6,7]. However, the phenotypic outcomes of ELOVL6 inhibition are highly context-dependent, dictated by both the genetic model and the dietary environment. In an atherogenic high-fat diet-induced MASH model, whole-body *Elovl6* knockout improves inflammation, oxidative stress, and fibrosis [6]. Conversely, in LKO mice, insulin sensitivity improves under HSD but shows minimal improvement under HFD [5]. Furthermore, hepatic ELOVL6 plays a unique role in maintaining bile acid homeostasis. Under lithogenic diet conditions, *Elovl6* deficiency carries a risk of promoting cholesterol crystallization and gallstone formation [55]. Therefore, liver-targeted ELOVL6 inhibition may be beneficial for MASH. However, its efficacy strictly depends on the patients’ metabolic status and active DNL flux.

### 5.2. Regulation of ELOVL6 Expression in White Adipose Tissue

The tissue specificity of ELOVL6 in white adipose tissue (WAT) is reflected not only in its role in DNL and lipid storage [11,56], but also in its impact on insulin signal transduction efficiency [4]. Under physiological conditions, it elongates C16:0-CoA to C18:0-CoA and subsequently contributes to MUFA synthesis. This process maintains fatty acid balance and is essential for ensuring lipid storage [53,57]. More specifically, ELOVL6 in WAT can also regulate insulin sensitivity through the HSL-ChREBP-ELOVL6 axis. Briefly, when HSL activity decreases, ChREBPα translocates into the nucleus and activates *ELOVL6* transcription. *ELOVL6* then increases the C18:1n-9 content in phospholipids and enhances membrane fluidity, thereby strengthening insulin signaling [4]. Furthermore, targeting *ELOVL6* in WAT may improve IR independently of obesity [1,11], but its effects need to be considered in coordination with other tissues such as muscle and the liver.

### 5.3. Regulation of ELOVL6 Expression in Brown Adipose Tissue

In brown adipose tissue (BAT), ELOVL6 is essential for full thermogenic recruitment upon cold exposure. BAT dissipates energy through non-shivering thermogenesis. It is an important metabolic tissue for maintaining body temperature and preventing obesity. Cold exposure induces changes in BAT fatty acid composition. These changes depend on ELOVL6 activity. *Elovl6* deficiency reduces the expression of mitochondrial electron transport chain components. This impairs thermogenic capacity [28]. However, this impairment can be offset by compensatory beiging of subcutaneous WAT. This suggests that ELOVL6 function in BAT is partially compensable. Therapeutically, the potential disruption of BAT thermogenic function by ELOVL6 inhibitors should be included in risk assessment.

### 5.4. Summary

In summary, targeting ELOVL6 in specific tissues, such as the liver and WAT, may improve insulin sensitivity and reduce lipotoxicity under certain dietary conditions, such as HSD. However, systemic inhibition still carries risks. These risks include impaired BAT thermogenesis and the potential for gallstone formation. Therefore, ELOVL6 should not be viewed as a universal therapeutic target. Instead, it should be considered a tissue- and context-dependent regulator.

## 6. ELOVL6 as a Potential Therapeutic Target for Metabolic Diseases

*ELOVL6* gene expression exhibits heterogeneity among different populations. In a Chinese Han population, the rs12504538 polymorphism in the *ELOVL6* gene was associated with an increased risk of T2DM [58]. In a southern Spanish population, genetic variations in *ELOVL6* were significantly associated with insulin sensitivity [59]. In a Mexican population, *ELOVL6* was strongly associated with clinical markers of multiple metabolic disorders [60]. Specific single nucleotide polymorphisms were linked to blood glucose levels and the degree of IR. Although genetic association studies suggest a link between *ELOVL6* polymorphisms and insulin sensitivity or T2DM risk in humans, these findings are primarily associative and lack interventional validation. The majority of current mechanistic evidence regarding the metabolic benefits of ELOVL6 inhibition comes from rodent models. Therefore, targeting ELOVL6 is currently still regarded as a therapeutic hypothesis. It is not yet an established clinical strategy.

Under an HFHS diet, *Elovl6*^−/−^ mice gain weight and develop hepatic steatosis to a similar extent as wild-type mice. Yet they exhibit improved insulin sensitivity [11]. This “fat but healthy” phenotype is called metabolically healthy obesity. It represents a potential therapeutic goal for ELOVL6-targeting drugs. However, there is no direct evidence that small-molecule ELOVL6 inhibitors can reproduce this phenotype. This may be because simply blocking ELOVL6 does not equate to restoring the physiological SFA/MUFA balance. Inhibiting fatty acid elongation alone may be insufficient to reverse established metabolic damage.

Dietary factors exert a bidirectional regulatory effect on ELOVL6 expression. A high-fructose diet activates the transcription factor ChREBP, which in turn activates *Elovl6* gene expression [51]. It drives the accumulation of long-chain fatty acids. This eventually leads to metabolic disorders. In contrast, an HFD, especially one rich in n-3 polyunsaturated fatty acids (PUFAs) like fish oil, suppresses hepatic *Scd1* and *Elovl6* expression [61]. It inhibits *ELOVL6* expression through nuclear receptor pathways. This improves insulin signaling. Furthermore, specific nutrients such as inositol can reduce HFD-induced histone H3/H4 acetylation. This decreases ChREBP binding to the *Elovl6* promoter. It thereby suppresses *Elovl6* transcription [51]. Compared with pharmacological interventions, dietary modifications are low-cost and highly accessible. They avoid the off-target effects and tissue-specific risks of drugs. Dietary interventions can also act synergistically with drug interventions. They enhance drug efficacy while reducing the drug dosage required to achieve the same treatment effect.

Furthermore, the unresolved molecular structure of ELOVL6 has historically limited structure-based rational drug design. Recently, the resolved structure of human ELOVL7 (PDB: 6Y7F) has provided a valuable surrogate template [27]. Using this as a template, a computational homology model of ELOVL6 can be generated, which facilitates the design of selective inhibitors targeting the conserved HxxHH catalytic motif and the substrate-binding tunnel. Nevertheless, it must be acknowledged that the residues forming the acyl-chain binding pocket and the TM6-TM7 loop are highly divergent between ELOVL6 and ELOVL7. Consequently, relying on these models may lead to inhibitors with poor selectivity or even unpredictable off-target effects. Therefore, future studies should still focus on resolving the experimental structure of ELOVL6, which will ultimately enable the development of more selective ELOVL6 inhibitors.

## 7. Conclusions

ELOVL6 contributes to the dynamic balance of the specified C16/C18 ratios in the indicated lipid pool and integrates nutritional signals such as insulin and glucose. It exerts specific regulatory roles in hepatic lipid storage, insulin signaling in adipose tissue, and pancreatic β-cell function. Meanwhile, its metabolic effects are highly dependent on dietary context and specific tissues. At the clinical level, ELOVL6 is a potential therapeutic target. However, drug development is limited by structural biology bottlenecks, tissue-specific risks, and the lack of stage-dependent intervention strategies.

Although significant progress has been made in ELOVL6 research, several key questions remain. First, the specific signaling mechanisms of C16 and C18 fatty acids in defined organelles, such as mitochondria and the endoplasmic reticulum, are not fully understood. Second, whether substrate overlap and functional compensation exist between ELOVL6 and other ELOVL family members needs further investigation. Future research should systematically explore the regulatory networks of ELOVL6 in different tissues. Specific ELOVL6-targeting drugs should be developed. Meanwhile, the possibility of co-targeting ELOVL6 with metabolic enzymes such as SCD1 should be investigated.

## Data Availability

No new data were created or analyzed in this study. Data sharing is not applicable to this article.

## Abbreviations

The following abbreviations are used in this manuscript:

AGPAT	1-acylglycerol-3-phosphate acyltransferase
Acyl-CoA	Acyl coenzyme A
Akt	Protein kinase B(PKB)
ALT	Alanine aminotransferase
ATF-3	Activating transcription factor 3
BAT	Brown adipose tissue
CerS4	Ceramide synthase 4
CerS6	Ceramide synthase 6
ChREBP	Carbohydrate-responsive element-binding protein
ChoRE	Carbohydrate response element
DAG	Diacylglycerol
DGAT	Diacylglycerol acyltransferase
DNL	De novo lipogenesis
ELOVL6	Elongation of very long chain fatty acids 6
ER	Endoplasmic reticulum
GPAT	Glycerol-3-phosphate acyltransferase
GSIS	Glucose-stimulated insulin secretion
HFD	High-fat diet
HFHS	High-fat/high-sucrose
HSD	high-sucrose diet
HSL	Hormone-sensitive lipase
I2PP2A	Protein phosphatase 2A inhibitor-2
Ldlr	Low density lipoprotein receptor
IL-1β	Interleukin-1β
LKO	liver-specific knockout
IR	Insulin resistance
IRS-1	Insulin receptor substrate-1
LPA	Lysophosphatidic acid
LXR	Liver X receptor
MASH	Metabolic dysfunction-associated steatohepatitis
MASLD	Metabolic dysfunction-associated steatotic liver disease
*Mlxip1*	MLX interacting protein-like
MUFA	Monounsaturated fatty acid
NLRP3	NLR family pyrin domain containing 3
PA	Phosphatidic acid
PKCε	Protein kinase C epsilon
PP2A	Protein phosphatase 2A
PUFA	Polyunsaturated fatty acid
SCD1	Stearoyl-CoA desaturase 1
SFA	Saturated fatty acid
SREBP-1c	Sterol regulatory element-binding protein-1c
SVLFA	Saturated very long-chain fatty acid
T2DM	Type 2 diabetes mellitus
TG	Triacylglycerol
VLCFA	Very long-chain fatty acid
VLC-PUFA	Very long-chain polyunsaturated fatty acid
WAT	White adipose tissue
